# Pembrolizumab-induced psoriasis and radiation recall: a shared immune pathway unveiled^؆^

**DOI:** 10.1016/j.abd.2026.501442

**Published:** 2026-08-17

**Authors:** Macarena Nougues, Paula Carolina Luna, Luciana Laura Tirelli, Margarita Larralde

**Affiliations:** Dermatology Department, Hospital Alemán de Buenos Aires, Buenos Aires, Argentina

Dear Editor,

A 40-year-old woman with no dermatologic history was diagnosed with stage IIa (cT2N0) triple-negative invasive ductal carcinoma of the left breast. She received neoadjuvant chemotherapy following the Keynote-522 protocol: carboplatin plus paclitaxel followed by doxorubicin and cyclophosphamide combined with pembrolizumab. She subsequently underwent breast-conserving surgery and adjuvant radiotherapy.

Two months after radiotherapy, she developed pruritic erythematous papules and plaques restricted to the irradiated breast (Fig. 1). A biopsy revealed a subcorneal neutrophilic pustule with mild acanthosis and focal spongiosis; PAS staining was negative, and no malignant cells were identified. Two weeks later, scaly erythematous annular plaques appeared on the trunk and limbs (Fig. 2 A‒B), while the breast lesion evolved to resemble these plaques (Fig. 3).

**Fig. 1:**
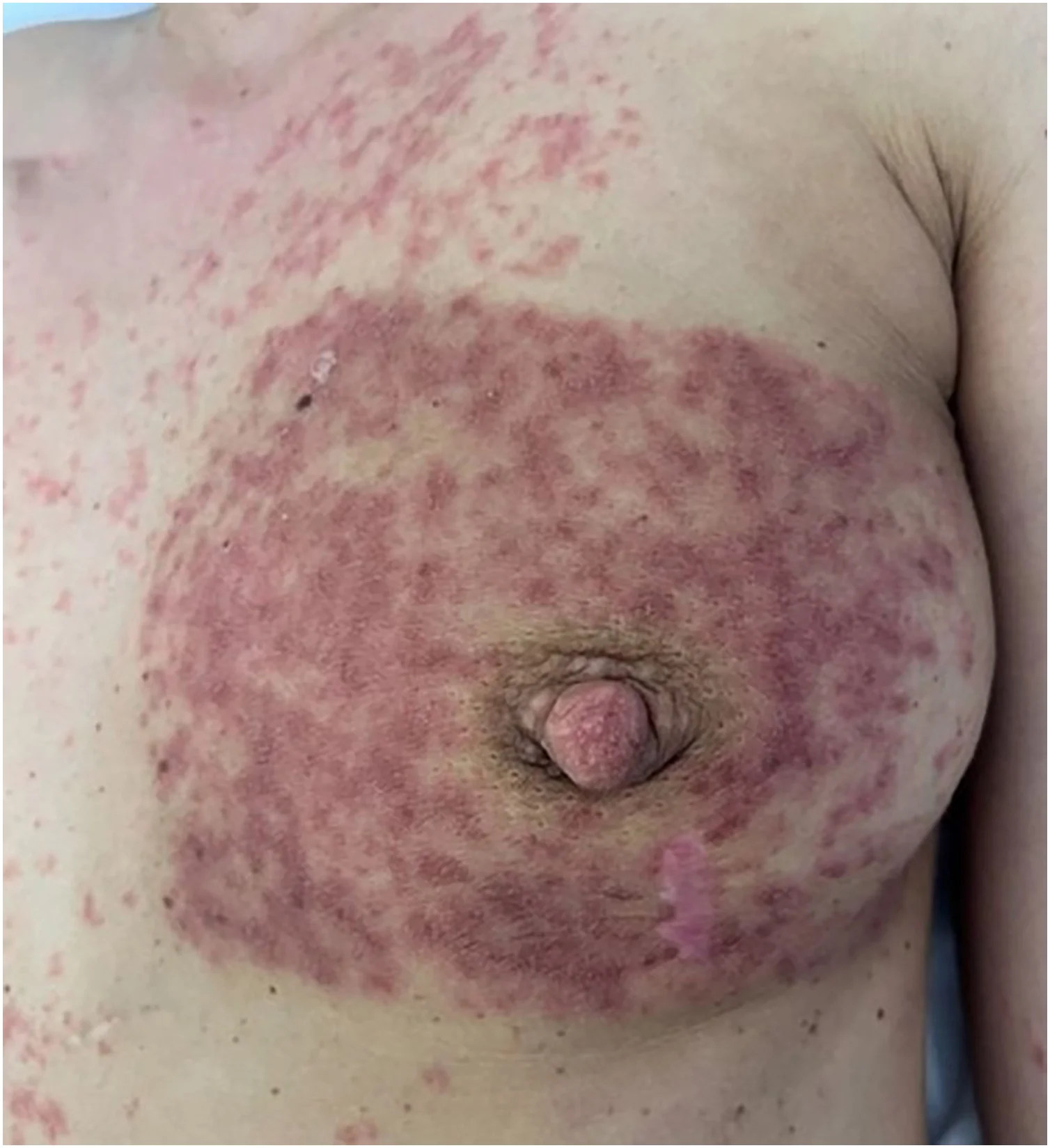
Well-demarcated, confluent, erythematous papules and plaques confined to the irradiated breast.

**Fig. 2:**
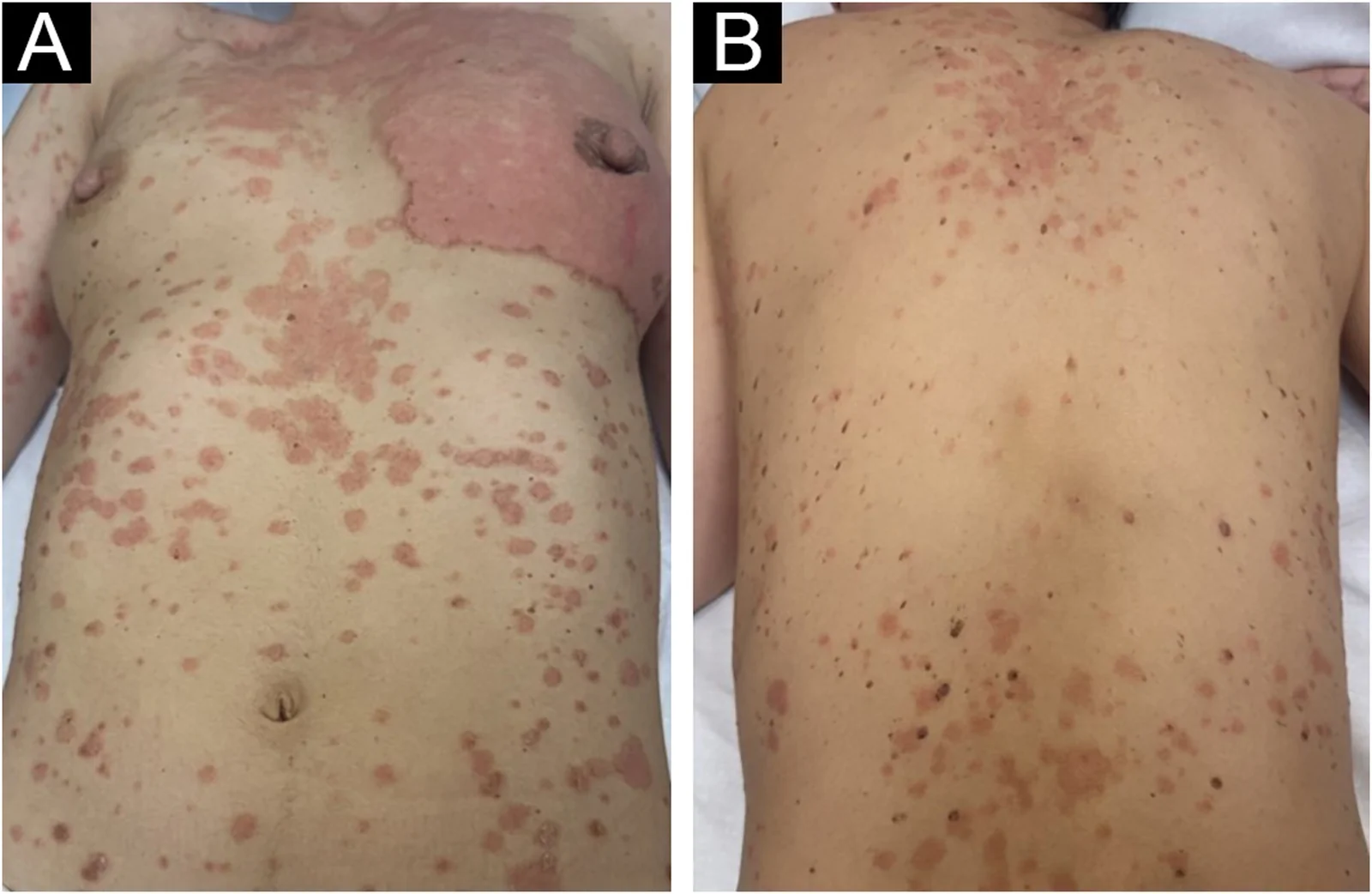
(A‒B) New scaly erythematous well-demarcated annular small plaques affecting the trunk and limbs.

**Fig. 3:**
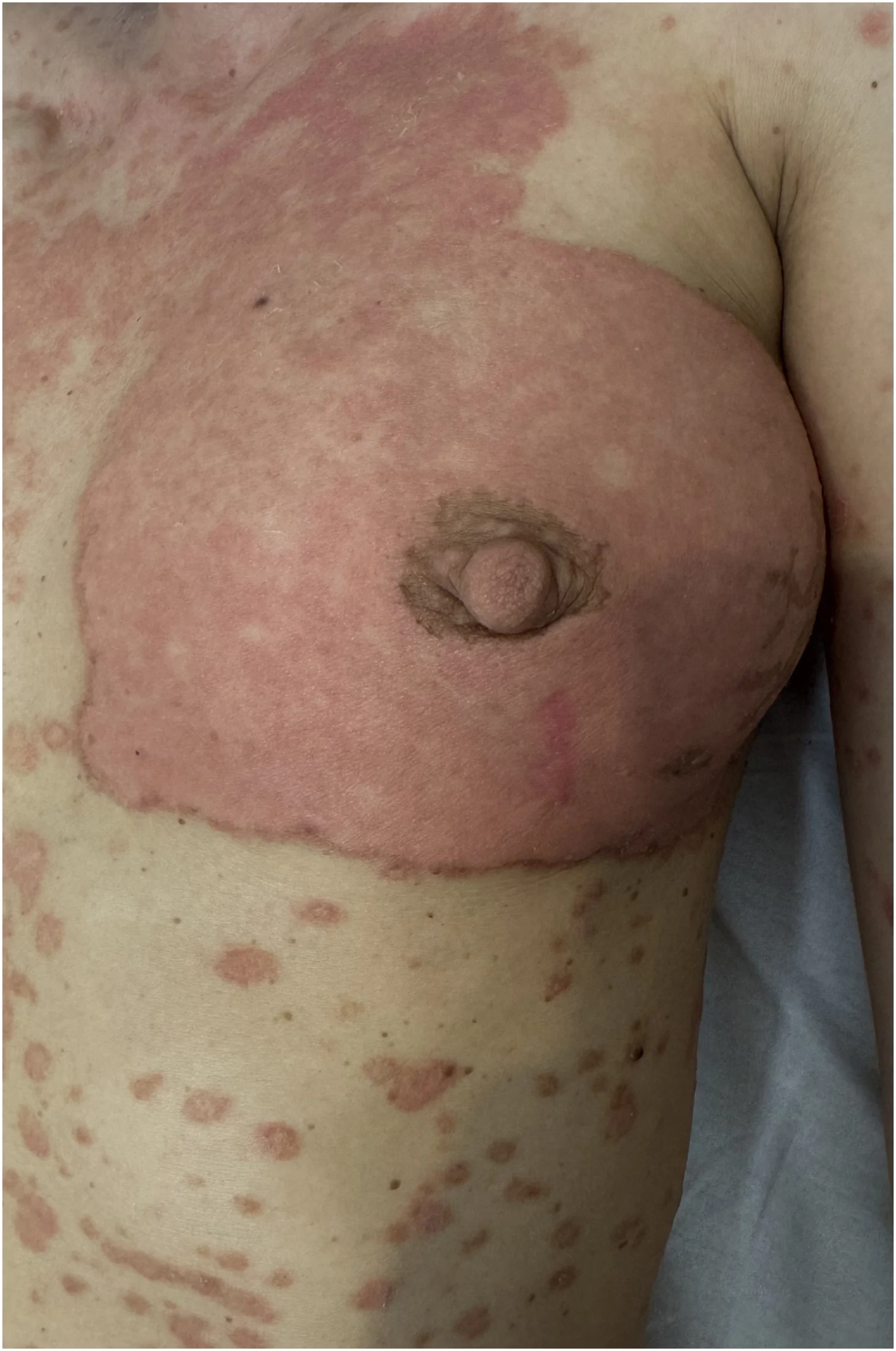
The breast lesion gradually faded into a milder phenotype that resembled the new plaques.

With clinical suspicion of psoriasis, two biopsies from the trunk and one from the leg demonstrated regular acanthosis, suprapapillary thinning, and neutrophilic microabscesses, consistent with psoriasiform dermatitis (Fig. 4 A‒B).

**Fig. 4:**
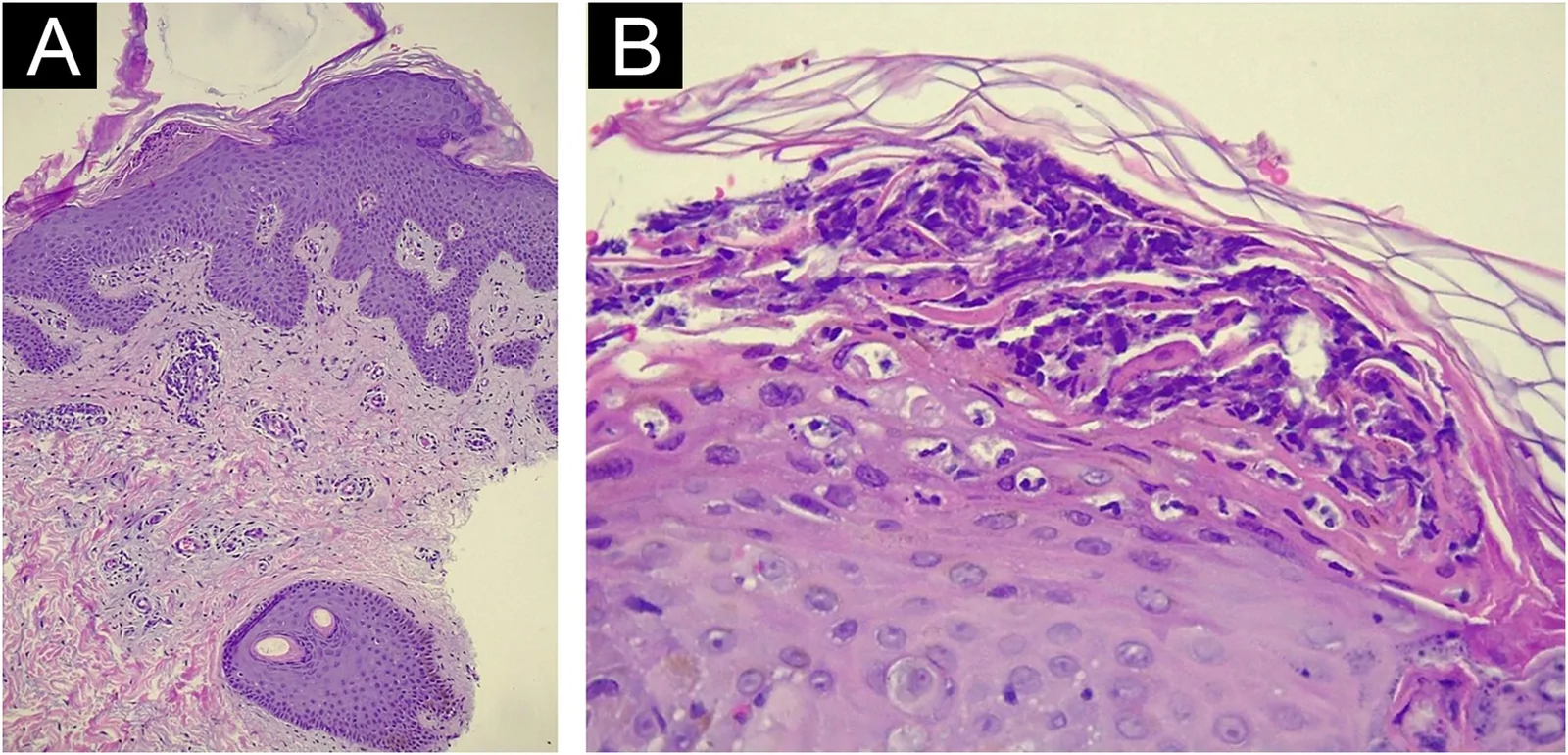
(A) Histopathology: Regular acanthosis, thinning of the suprapapillary plates, and neutrophilic microabscesses in the stratum corneum, findings consistent with psoriasiform dermatitis. (B) Figure A at higher magnification (Hematoxylin & eosin, x 100, x 400).

Considering the presentation pattern, oncologic history, and sequential evolution ‒ from a localized eruption in the irradiated field to a generalized psoriasiform eruption ‒ the findings supported a diagnosis of pembrolizumab-induced psoriasis initially presenting as radiation recall dermatitis. The early presence of a subcorneal neutrophilic pustule reinforces this interpretation, reflecting the neutrophilic pattern characteristic of Immune Checkpoint Inhibitor (ICI)-related psoriasiform reactions. These findings suggest pembrolizumab as the most likely trigger of both phenomena, supporting the term “radiation recall psoriasis” as an analog of “radiation recall dermatitis” with a psoriasiform pattern.

Cutaneous immune-related Adverse Events (irAEs) are among the most frequent toxicities of ICIs, occurring in up to 70% of patients.^1^ Psoriasis develops in approximately 3%–5%, either de novo or as a flare of pre-existing disease.^2^ Pembrolizumab is strongly associated with psoriasiform reactions, as PD-1 blockade promotes Th1/Th17 activation and proinflammatory cytokine production, leading to characteristic histopathologic features.^3^

Radiation recall dermatitis is an inflammatory reaction confined to previously irradiated skin following exposure to a systemic agent. It is most commonly associated with chemotherapeutics and rarely with ICIs.^4^ Latency ranges from days to years and is thought to involve persistent local sensitization within an “immunocompromised district”.^5^

Although pembrolizumab was considered the main trigger, previously administered agents such as paclitaxel and doxorubicin have also been associated with radiation recall. However, the temporal pattern in this case favors pembrolizumab, as the eruption occurred after prior exposure during the neoadjuvant phase, within a latency consistent with irAEs. While the contribution of prior chemotherapeutics cannot be excluded, the delayed onset and the known immunomodulatory effects of PD-1 blockade make pembrolizumab the most plausible trigger. Additionally, the initial biopsy, although nonspecific, showed features such as a subcorneal neutrophilic pustule that may relate to the psoriasiform pattern observed later. This clinicopathologic evolution supports a shared pharmacologically triggered immune mechanism underlying both the radiation recall reaction and the generalized eruption.

A Koebner-like phenomenon could be considered; however, the initial strict localization to the irradiated field followed by generalization, together with the immunomodulatory effects of PD-1 blockade, favors a radiation recall-related mechanism.

From a differential standpoint, the initial eruption could theoretically resemble a Wolf Isotopic Response (WIR). However, this hypothesis is less consistent with its defining criteria. By definition, WIR denotes the occurrence of a new, unrelated dermatosis at the site of a previously healed cutaneous disease ‒ most often herpes zoster or lichen planus ‒ and the resolution of the primary dermatosis is considered a *sine qua non* condition for diagnosis.^6^ In this case, the antecedent condition was radiation injury rather than a healed dermatosis, and the subsequent psoriasiform eruption later extended beyond the irradiated field. Moreover, WIR typically arises without a pharmacologic trigger, whereas this eruption followed re-exposure to pembrolizumab, a PD-1 inhibitor known to activate Th1/Th17-mediated inflammation. Therefore, the initial strict confinement to the irradiated area, temporal latency, and dependence on a systemic trigger are more consistent with a radiation recall dermatitis or psoriasis mechanism than with a true isotopic phenomenon.^7^

Management of ICI-induced psoriasis aims to control cutaneous disease while preserving antitumor efficacy. Continuation of immunotherapy is generally recommended, with topical agents or non-steroidal systemic therapies. In moderate to severe cases, apremilast, acitretin, methotrexate, or biologics targeting IL-17/IL-23 may be safely used without compromising oncologic response.^8^ For radiation recall dermatitis, corticosteroids are the mainstay, and discontinuation of the triggering agent is rarely required.

Importantly, the emergence of dermatologic irAEs, including psoriasis, has been correlated with improved overall survival in several cancer types, suggesting that skin inflammation may reflect effective systemic immune activation rather than treatment failure.^9^

This case highlights the importance of early dermatologic evaluation in patients receiving immunotherapy, as clinicopathologic correlation helps avoid misdiagnosis and unnecessary treatment interruption. To our knowledge, this represents the first report of pembrolizumab-induced de novo psoriasis presenting with radiation recall dermatitis, supporting a shared pathogenic mechanism. Early recognition allows continuation of immunotherapy and favorable outcomes.

## Authors’ contributions

Macarena Nougues: Manuscript critical review; preparation and writing of the manuscript.

Luciana Laura Tirelli: Manuscript critical review; preparation and writing of the manuscript.

Paula Carolina Luna: Approval of the final version of the manuscript.

Margarita Larralde: Approval of the final version of the manuscript.

## Financial support

None declared.

## Research data availability

The entire dataset supporting the results of this study was published in this article.

## Conflicts of interest

None declared.
